# Identification of somatic mutations of the *MEN1* gene in sporadic endocrine tumours

**DOI:** 10.1054/bjoc.2000.1385

**Published:** 2000-10

**Authors:** L Bergman, C Boothroyd, J Palmer, S Grimmond, M Walters, B Teh, J Shepherd, L Hartley, N Hayward

**Affiliations:** Queensland Cancer Fund Research Unit, Joint Experimental Oncology Programme of the Queensland Institute of Medical Research and the University of Queensland, Herston, QLD 4029, Australia; Department of Obstetrics and Gynaecology, Royal Women’s Hospital, Post Office Royal Brisbane Hospital, Herston, QLD 4029, Australia; Department of Clinical Genetics, Karolinska Institute, Stockholm, Sweden; Department of Surgery, University of Tasmania, Hobart, TAS, 7000, Australia; 79 Wickham Terrace, Brisbane, QLD 4000, Australia

**Keywords:** MEN 1, LOH, mutation analysis, endocrine tumour, sporadic

## Abstract

Endocrine tumours of the pancreas, anterior pituitary or parathyroids arise either sporadically in the general population, or as a part of inherited syndromes such as multiple endocrine neoplasia type 1 (MEN 1). The mechanisms responsible for the development of sporadic endocrine lesions are not well understood, although loss of heterozygosity (LOH) of the *MEN1* locus on chromosome 11q13 and somatic mutation of the *MEN1* gene have been frequently associated with the development of MEN 1-type sporadic endocrine lesions. To further investigate the role of the *MEN1* gene in sporadic endocrine tumorigenesis, we analysed DNA from 14 primary parathyroid lesions, 8 anterior pituitary tumours and 3 pancreatic tumours for the presence of somatic *MEN1* gene mutations and LOH of seven microsatellite markers flanking the *MEN1* locus. In addition, we similarly analysed 8 secondary parathyroid lesions which arose in patients with chronic renal failure. None of the patients studied had a family history of MEN 1. Three primary parathyroid lesions and one pancreatic tumour (glucagonoma) were found to have lost one allele at the *MEN1* locus. Somatic mutations were identified by SSCP and sequence analysis in one of these parathyroid lesions (P320L) and in the glucagonoma (E179V). These results support previous findings that inactivation of the *MEN1* tumour suppressor gene contributes to the development of sporadic MEN 1-type endocrine lesions but is not associated with the development of parathyroid hyperplasia seen in some renal failure patients. © 2000 Cancer Research Campaign


British Journal of Cancer (2000) 83(8), 1003–1008
© 2000 Cancer Research Campaign
doi: 10.1054/ bjoc.2000.1385, available online at http://www.idealibrary.com on IDEAL®

### Identification of somatic mutations of the *MEN1* gene in sporadic endocrine tumours

**L Bergman, C Boothroyd[1], J Palmer, S Grimmond, M Walters, B Teh[2,3], J Shepherd[3], L Hartley[4] and N Hayward**

Queensland Cancer Fund Research Unit, Joint Experimental Oncology Programme of the Queensland Institute of Medical Research and the University of Queensland, Herston QLD 4029, Australia; [1]Department of Obstetrics and Gynaecology, Royal Women's Hospital, Post Office Royal Brisbane Hospital, Herston QLD 4029, Australia; [2]Department of Clinical Genetics, Karolinska Institute, Stockholm, Sweden; [3]Department of Surgery, University of Tasmania, Hobart, TAS 7000 Australia; [4]79 Wickham Terrace, Brisbane QLD 4000, Australia

**Summary** Endocrine tumours of the pancreas, anterior pituitary or parathyroids arise either sporadically in the general population, or as a part of inherited syndromes such as multiple endocrine neoplasia type 1 (MEN 1). The mechanisms responsible for the development of sporadic endocrine lesions are not well understood, although loss of heterozygosity (LOH) of the *MEN1* locus on chromosome 11q13 and somatic mutation of the *MEN1* gene have been frequently associated with the development of MEN 1-type sporadic endocrine lesions. To further investigate the role of the *MEN1* gene in sporadic endocrine tumorigenesis, we analysed DNA from 14 primary parathyroid lesions, 8 anterior pituitary tumours and 3 pancreatic tumours for the presence of somatic *MEN1* gene mutations and LOH of seven microsatellite markers flanking the *MEN1* locus. In addition, we similarly analysed 8 secondary parathyroid lesions which arose in patients with chronic renal failure. None of the patients studied had a family history of MEN 1. Three primary parathyroid lesions and one pancreatic tumour (glucagonoma) were found to have lost one allele at the *MEN1* locus. Somatic mutations were identified by SSCP and sequence analysis in one of these parathyroid lesions (P320L) and in the glucagonoma (E179V). These results support previous findings that inactivation of the *MEN1* tumour suppressor gene contributes to the development of sporadic MEN 1-type endocrine lesions but is not associated with the development of parathyroid hyperplasia seen in some renal failure patients. © 2000 Cancer Research Campaign

**Keywords:** MEN 1; LOH; mutation analysis; endocrine tumour; sporadic

Endocrine tumours predominantly arise sporadically in the general population, but sometimes occur as part of inherited endocrine neoplasia syndromes. Multiple endocrine neoplasia type 1 (MEN 1) is an autosomal dominant disorder characterized primarily by hyperplasia and/or neoplasia of the parathyroid glands, anterior pituitary and endocrine pancreas. The *MEN1* gene is a tumour suppressor gene located at chromosome band 11q13 (Larsson et al, 1988) and was recently identified by positional cloning (Chandrasekharappa et al, 1997).

As well as its involvement in familial MEN 1 and related syndromes, the *MEN1* locus has also been implicated in the development of MEN 1-type sporadic endocrine tumours. Loss of heterozygosity (LOH) of markers flanking the *MEN1* gene has been reported in sporadic primary parathyroid lesions (Tahara et al, 1996; Farnebo et al, 1997a), pancreatic tumours (Teh et al, 1990; Debelenko et al, 1997a), and anterior pituitary tumours (Bates et al, 1997; Dong et al, 1997), as well as in sporadic cases of tumours less commonly associated with MEN 1, such as lung, thymic and gastric carcinoids (Jakobovitz et al, 1996; Debelenko et al, 1997b), lipomas (Dong et al, 1997) and cutaneous tumours (Pack et al, 1997). Mutations in the *MEN1* gene have been detected in all of these tumour types (Heppner et al, 1997; Debelenko et al, 1997c; Zhuang et al, 1997a; Zhuang et al, 1997b; Böni et al, 1998; Vortmeyer et al, 1998).

This study aims to continue investigations into the role of the *MEN1* gene in sporadic endocrine tumorigenesis, by screening for LOH of 11q13 markers and mutations in the *MEN1* gene in a series of sporadic tumours of the pancreas, pituitary and parathyroids. In addition, we aim to investigate the role of the *MEN1* locus in the development of parathyroid lesions that have arisen secondarily due to hypercalcaemia following renal transplantation.

### MATERIALS AND METHODS

### Patients and tumours

After obtaining informed consent, endocrine lesions from 33 patients undergoing surgery to remove pancreatic ($n = 3$), anterior pituitary ($n = 8$) or parathyroid lesions ($n = 22$) at the Royal Brisbane or Princess Alexandra Hospitals in Brisbane were included in the study. A peripheral blood sample for germline DNA analysis was also acquired from each patient, for which they gave informed consent. Diagnosis of primary hyperparathyroidism was based on the following criteria: elevated serum calcium (>2.6 mmol/l) and/or serum parathyroid hormone (>5.5 μmol/l); and presence of hyperplasia or adenoma following histological analysis of frozen sections of parathyroid tissue. Usually only one of the four parathyroid glands was affected in these patients. Secondary hyperparathyroidism arising in patients with chronic (or end stage) renal failure is associated with hyperplasia of all

*Received 23 August 1999*
*Revised 2 May 2000*
*Accepted 15 June 2000*

*Correspondence to:* N Hayward four parathyroid glands. A diagnosis of secondary hyperparathyroidism was made in 8 patients, 6 of whom also underwent (successful) renal transplants. In general, any hyperplastic material remaining after diagnostic histopathological studies was analysed. A diagnosis of glucagonoma was made in a patient with elevated fasting plasma glucagon levels (>100 ng/l) and positive staining for glucagon in an islet cell tumour. One case of insulinoma was diagnosed in a hypoglycaemic patient with an insulin-positive islet cell tumour. Pituitary tumours were classified as growth hormone- or prolactin-producing according to clinical evidence of hormone excess, and the remaining tumours were classified as non-functioning. A detailed clinical and family history of each patient was taken to eliminate the possibility of MEN 1 or familial isolated hyperparathyroidism.

### Isolation of DNA

Constitutional DNA was isolated from lymphoblastoid cell lines (LCLs) using a method based on that of Miller et al (1986). DNA was isolated from frozen tumour tissue as follows: surrounding stromal tissue was removed from the specimen with a scalpel, then tumorous material was homogenized in lysis buffer (10 mM Tris-HCl, 2 mM EDTA, 400 mM NaCl) using an omni-mixer (DuPont Sorvall), and DNA was isolated using the same protocol as for LCLs.

### SSCP analysis

Tumour samples were screened for the presence of somatic mutations using single strand conformation polymorphism (SSCP) analysis (Orita et al, 1989). Exons 3–10 of the *MEN1* gene were amplified by PCR using approximately 50 ng of tumour DNA from each patient in a 10 µl reaction volume as follows: 10 mM Tris-HCl, 50 mM KCl, 1.5 mM $MgCl_2$, 200 µM dNTPs (Promega), 10 pmol each primer (listed in Lemmens et al, 1997), 5% DMSO, 0.75 U AmpliTaq Gold DNA polymerase (PE Biosystems) and 1 µCi [$\alpha$-$^{32}$P]-dCTP (Amersham). Samples were cycled in an Omnigene (Hybaid) thermal cycler as follows: 95°C for 12 minutes, followed by 35 cycles of 95°C for 1 minute, 62°C (64°C for gene fragment 10.1) for 1 minute and 72°C for 90 seconds. 20 µl of loading dye were added to each PCR product, and 2.5 µl of each sample were electrophoresed under two different gel conditions: (i) 0.6 × MDE gel solution (FMC bioproducts) at 5 W for 16 hours, and (ii) 0.5 × MDE containing 10% glycerol at 7 W for 16 hours. Gels were exposed to X-ray film (Fuji) overnight. Autoradiographs were examined for samples showing shifts in migration patterns compared with known wild type controls.

### LOH analysis

Approximately 50 ng of LCL DNA and corresponding tumour DNA from each patient were amplified by PCR using the following microsatellite markers.

#### *D11S4936, D11S4939, D11S4940, D11S449*

These markers were amplified as described by Manickam et al (1997), using 0.75 U of AmpliTaq Gold DNA polymerase. Primer sequences for D11S449 were obtained from Debelenko et al (1997d).

#### *PYGM (CAGA$_n$ repeat)*

PCR reactions were prepared as described for SSCP analysis and cycled as follows: 95°C for 12 minutes, followed by 25 cycles of 95°C for 1 minute, 62°C for 1 minute and 72°C for 2 minutes. Primer sequences were obtained from Iwasaki et al (1992).

#### *D11S480, D11S913*

PCR reactions were prepared as described for SSCP analysis and cycled as follows: 95°C for 12 minutes, followed by 35 cycles of 95°C for 1 minute, 58°C (53°C for D11S913) for 1 minute and 72°C for 90 seconds. Primer information was obtained from Debelenko et al (1997d).

Twenty µl of loading dye were added to PCR products and 3.5 µl of each sample were electrophoresed through a 6% denaturing polyacrylamide (19:1) gel containing 7 M urea at 1500 V for 3–6 hours, depending on product size. Gels were exposed to autoradiographic film (Fuji) for 1 hour overnight. Heterozygous samples were examined by eye for a reduced intensity of one allele in the tumour DNA lane, compared with the adjacent normal DNA lane. Constitutionally homozygous samples were scored as non-informative.

### DNA sequence analysis

Approximately 50 ng of DNA to be analysed were amplified by PCR as described for SSCP, but in a 50 µl reaction volume without radiolabel. Exon 2 of the *MEN1* gene was amplified using the following primers: (forward) 5′-GTGAGCAGAGGCTGAAGAGG-3′ and (reverse) 5′-ATAACACCTGCCGAACCTCAC-3′ with an annealing temperature of 64°C. 1 M betaine was included in this reaction instead of DMSO. The following primers were used for PCR of exon 3: 5′-AGGTTGGGTCACAGGCTTG-3′ (forward) and 5′-CTATGTGGGTGGTGATGGG-3′ (reverse) and annealed at 58°C. PCR products were purified by agarose gel electrophoresis and bands of the correct size were excised. DNA was isolated from the agarose using a QIAquick gel extraction kit (QIAGEN) as per the manufacturer’s instructions. 200–300 ng of purified PCR product was sequenced using Big Dye dye terminator reaction premix (PE Biosystems) as per the manufacturer’s instructions in a half reaction volume. Primers used were the same as for PCR. Cycling reactions were performed on a Selby TS-MP96 thermal cycler. Sequences were determined using an ABI377 automated sequencer, and sequence traces were manually analysed for the presence of heterozygous peaks. In addition, sequence traces were aligned to the *MEN1* gene sequence (GenBank U93237) using Mac Vector 4.1.1 (Kodak) software to identify possible homozygous base changes.

### RESULTS

A total of 8 sporadic pituitary tumours, 3 pancreatic tumours, 14 primary parathyroid lesions and 8 secondary parathyroid lesions were examined for the presence of allelic deletions of the *MEN1* locus on chromosome 11q13 and mutations of the *MEN1* gene. Clinical details of these tumours are given in Tables 1 and 2.

LOH of microsatellite markers located within an approximately 2.8 megabase (Mb) region flanking the *MEN1* locus, between D11S480 and D11S1337 (Manickam et al, 1997), was detected in 4/33 sporadic endocrine lesions – 1 glucagonoma and 3 primary parathyroid lesions (Tables 1 and 2). Figure 1 shows characteristic LOH patterns detected for each of the 7 markers used.

 © 2000 Cancer Research Campaign

**Table 1** Clinical features and LOH analysis of 22 sporadic parathyroid lesions

| Patient ID No. | Age at surgery (years) | 1° or 2° | Glands involved | Clinical details | D11S480 | PYGM | D11S4940 | D11S4939 | D11S4936 | D11S449 | D11S913 |
|---|---|---|---|---|---|---|---|---|---|---|---|
| 40651 | 82 | 1° | R sup | adenoma | ● | ● | – | – | – | ● | – |
| 40652 | 59 | 1° | R sup | adenoma | ○ | ○ | – | – | – | ○ | ○ |
| 40654 | 82 | 1° | L inf | adenoma | ● | ● | ● | – | ● | ● | ● |
| 40673 | 44 | 1° | 1 gland | adenoma | – | ○ | – | – | – | ○ | ○ |
| 40703 | 24 | 1° | L inf | adenoma | ○ | ○ | – | ○ | ○ | ○ | – |
| 40712 | 24 | 1° | R sup | adenoma | – | ○ | ○ | ○ | – | – | ○ |
| 40732 | 49 | 1° | L sup, R sup | hyperplasia | ○ | ○ | – | ○ | ○ | ○ | ○ |
| 40734 | 47 | 1° | L sup | hyperplasia | ○ | | – | – | – | – | – |
| 40744 | 36 | 1° | R inf | adenoma | ○ | – | ○ | – | ○ | ○ | ○ |
| 40767 | 50 | 1° | R sup | adenoma | ○ | ○ | ○ | – | – | – | – |
| 40769 | 49 | 1° | R inf, R sup | adenoma | – | ● | ● | ● | ● | ● | – |
| 40776 | 67 | 1° | R inf | adenoma; diabetes, mild renal impairment | ○ | ○ | ○ | ○ | ○ | ○ | ○ |
| 40795 | 82 | 1° | R inf | adenoma | – | ○ | ○ | – | – | – | ○ |
| 70006 | | 1° | | | ○ | ○ | ○ | – | ○ | ○ | – |
| 40714 | 44 | 2° | all 4 | renal transplant | – | – | – | ○ | ○ | ○ | ○ |
| 40715 | 64 | 2° | all 4 | renal transplant | ○ | ○ | – | ○ | ○ | ○ | ○ |
| 40718 | 48 | 2° | all 4 | renal transplant | ○ | ○ | ○ | – | – | ○ | ○ |
| 40719 | 36 | 2° | all 4 | renal transplant | ○ | – | – | ○ | – | ○ | – |
| 40723 | 44 | 2° | all 4 | renal transplant | ○ | ○ | – | ○ | ○ | ○ | ○ |
| 40724 | 53 | 2° | all 4 | renal transplant | ○ | ○ | ○ | ○ | ○ | ○ | ○ |
| 40726 | 62 | 2° | all 4 | diabetes, nephropathy | ○ | ○ | ○ | ○ | ○ | ○ | ○ |
| 40808 | | 2° | all 4 | renal failure | ○ | ○ | ○ | ○ | – | ○ | – |

1° = primary hyperparathyroidism; 2° = secondary hyperparathyroidism; L = left; R = right; sup = superior; inf = inferior; ○ = no LOH; ● = LOH; – = not informative; blanks indicate information unavailable

**Table 2** Clinical features and LOH analysis of sporadic pancreatic and anterior pituitary lesions

| Patient ID No. | Age at surgery (years) | Tumour type | Hormone over-secreted | Symptoms | D11S480 | PYGM | D11S4940 | D11S4939 | D11S4936 | D11S449 | D11S913 |
|---|---|---|---|---|---|---|---|---|---|---|---|
| 40653 | 70 | pancreas | gluc | | ● | ● | ● | – | – | – | ● |
| 40711 | 82 | pancreas | ins | hypoglycaemia | ○ | ○ | ○ | ○ | ○ | ○ | – |
| 41042 | 51 | pancreas | ins | | ○ | ○ | ○ | ○ | – | – | – |
| 40716 | 57 | pituitary | NF | | ○ | ○ | ○ | ○ | – | ○ | ○ |
| 40717 | 59 | pituitary | NF | hypopituitarism | – | ○ | – | – | – | ○ | – |
| 40741 | 76 | pituitary | NF | | – | – | – | ○ | – | ○ | ○ |
| 40768 | 50 | pituitary | pro | | ○ | ○ | ○ | ○ | – | ○ | ○ |
| 40773 | 31 | pituitary | pro | galactorrhoea, headaches | ○ | ○ | – | ○ | – | ○ | – |
| 40785 | 39 | pituitary | GH | acromegaly, headaches | ○ | ○ | ○ | – | – | ○ | – |
| 40788 | 27 | pituitary | NF | | ○ | ○ | ○ | ○ | ○ | ○ | ○ |
| 40819 | | pituitary | GH | | ○ | – | ○ | – | ○ | ○ | |

gluc = glucagon; ins = insulin; GH = growth hormone; pro = prolactin; NF = clinically non-functioning; ○ = no LOH; ● = LOH; – = not informative; blanks indicate information unavailable

Exons 3–10 of the *MEN1* gene were analysed for the presence of somatic mutations in all 33 sporadic tumours using SSCP. Band-shifts (Fig. 2) were detected in the glucagonoma sample and in one of the parathyroid lesions also showing LOH. Sequencing of the relevant exons resulted in the identification of 2 missense mutations, in exons 3 and 7 respectively. Patient 40653–001 was found to have a 4387A>T base change, resulting in a protein change of E179V, and the mutation in patient 40769–001 was 6071C>T (P320L). The P320L mutation has been previously identified in a Japanese MEN 1 patient (Tanaka et al, 1997), but the E179V mutation is novel. The corresponding constitutional DNA from these patients was found to be wild type, thus confirming that the mutations occurred somatically. Sequence traces demonstrating this are shown in Figure 3.

In addition to SSCP analysis of the other two parathyroid adenomas displaying LOH, the entire coding region of the *MEN1* gene was sequenced in these samples. No mutation was detected in DNA from these tumours.

During the process of mutation analysis, a number of sequence polymorphisms were detected. These include the commonly-reported D418D and R171Q variants, as well as an A342A polymorphism (6138G>A), which we report here for the first time. This polymorphism was only identified once out of approximately 100 MEN 1 patient samples and sporadic endocrine tumour sample analysed, and was found in the tumour sample with the P320L mutation. For these reasons, and also because the protein sequence is not altered, this base change was not deemed to be a somatic mutation.

*© 2000 Cancer Research Campaign*

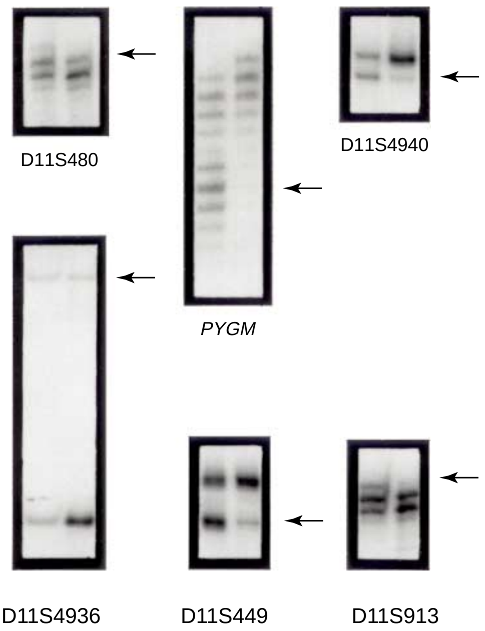

**Figure 1** Representative patterns of LOH detected for 6 of the 7 microsatellite markers analysed. For each marker, lane 1 is LCL DNA, and lane 2 is corresponding tumour DNA. The PYGM tumour lane also shows possible microsatellite instability

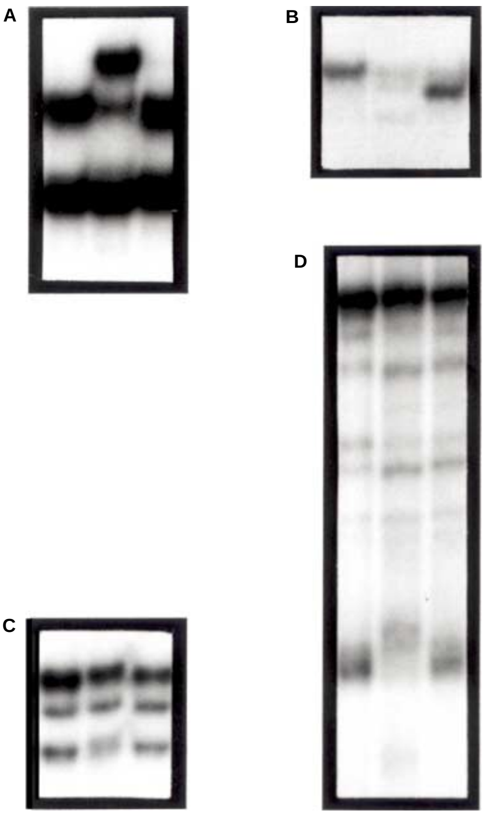

**Figure 2** Autoradiographs showing SSCP band-shifts for 2 somatic mutations. (**A**) Migration pattern for exon 3.1 in 0.6 X MDE. Lane 1 is wild type, lane 2 is the E179V missense mutation, and lane 3 contains a sample carrying a R171Q polymorphism. (**B**) Migration pattern for exon 3.1 in a gel containing 10% glycerol, loaded as per. **A**. (**C**) Exon 7 PCR products in 0.6 X MDE. Lanes 1 and 3 are wild type, and lane 2 contains the P320L missense mutation. (**D**) The same samples as in **C**, in a gel containing 10% glycerol.

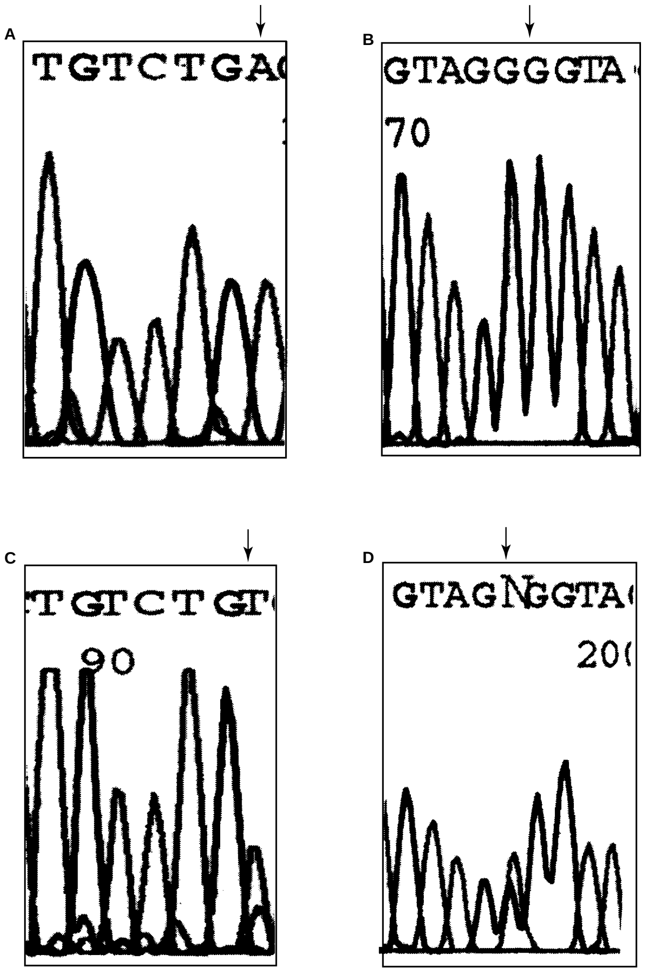

**Figure 3** Sequence traces showing somatic *MEN1* gene mutations identified. (**A**) A portion of exon 3 sequence in LCL and (**B**) same sequence in the corresponding glucagonoma showing A–T transversion responsible for E179V. (**C**) Portion of exon 7 complementary strand from LCL and (**D**) in corresponding parathyroid adenoma showing G–A (reverse strand) transition resulting in a P320L substitution.

### DISCUSSION

The role of the *MEN1* gene in sporadic endocrine tumorigenesis is well established. To further analyse the extent to which this gene is involved in the development of such tumours, we analysed 14 primary and 8 secondary parathyroid lesions, 3 pancreatic tumours and 8 pituitary tumours for the presence of allelic deletions and somatic mutations.

Of the 14 primary parathyroid lesions analysed, 3 (21%) showed LOH at the *MEN1* locus, and 1 (7%) of these carried a somatic mutation. Previous studies have found the rate of allelic deletion of the *MEN1* locus in this type of tumour to be similar, at approximately 30% (Carling et al, 1998; Farnebo et al, 1998). Moreover, *MEN1* gene mutations have previously been identified in 9–21% of parathyroid lesions studied (Heppner et al, 1997; Shan et al, 1998), comparable with our results. Parathyroid tumorigenesis can occur by other mechanisms such as cyclin D1 overexpression (Rosenberg et al, 1991), however this possibility was not assessed in this study. Of the 8 secondary parathyroid lesions examined, no LOH or *MEN1* gene mutations were detected. This

*© 2000 Cancer Research Campaign*

result supports those of previous studies (Farnebo et al, 1997a,b), suggesting that hyperparathyroidism arising as a consequence of renal failure occurs following some other genetic trigger.

Of the three pancreatic adenomas analysed, a glucagonoma was found to have both LOH and a somatic *MEN1* mutation. The low number of such lesions precludes statistical comparisons between this and other studies. No LOH or mutations were detected in any of the pituitary tumour samples. Once again, a relatively small number of samples was analysed, but previous studies reflect a similar finding, wherein 10% or fewer pituitary tumours show involvement of the *MEN1* locus (Zhuang et al, 1997b; Prezant et al, 1998). Numerous other loci have been implicated in pituitary tumorigenesis, including the *gsp* oncogene (Landis et al, 1989), and the *TP53* (Bates et al, 1997) and *CDKN2A* (Woloschak et al, 1996) tumour suppressor genes.

In the four tumours with LOH, allelic loss was detected across the entire region studied. This confirms the findings of others, in which deletions of the wild type allele in sporadic endocrine tumours are generally large and cover many kilobases (Heppner et al, 1997). Since some of the tumours analysed were uninformative at the microsatellite markers closest to the *MEN1* gene (e.g. PYGM and D11S4940), it is formally possible that some small allelic deletions might have gone undetected. However, because large deletions are usually observed, this is unlikely. It is possible that the two tumours carrying LOH at the *MEN1* locus without detectable *MEN1* gene mutations have the second *MEN1* allele inactivated by some other mechanism such as transcriptional silencing by promoter methylation, although we were not able to test this in this study, as RNA was not available from these tumours.

In summary, we have found LOH at the *MEN1* locus in 3 sporadic primary parathyroid lesions and in 1 sporadic glucagonoma. Somatic mutations in the *MEN1* gene were identified in one of these parathyroid lesions and in the glucagonoma. This supports previous findings that the *MEN1* gene is involved in the development of a subset of sporadic MEN 1-type endocrine lesions. Also confirmed are previous results suggesting that the development of secondary hyperparathyroidism occurs by some other genetic mechanism, rather than mutation of the *MEN1* gene.

### ACKNOWLEDGEMENTS

We are grateful to the Queensland Cancer Fund and the Australian National Health and Medical Research Council for supporting this research. We also thank the patients for their participation.

### REFERENCES

Bates AS, Farrell WE, Bicknell EJ, McNicol AM, Talbot AJ, Broome JC, Perrett CW, Thakker RV and Clayton RN (1997) Allelic deletion in pituitary adenomas reflects aggressive biological activity and has potential value as a prognostic marker. *J Clin Endocrinol Metab* **82**: 818–824

Böni R, Vortmeyer AO, Pack S, Park W-S, Burg G, Hofbauer G, Darling T, Liotta L and Zhang Z (1998) Somatic mutations of the MEN1 tumor suppressor gene detected in sporadic angiofibromas. *J Invest Dermatol* **111**: 539–540

Carling T, Correa P, Hessman O, Hedberg J, Skogseid B, Lindberg D, Rastad J, Westin G and Akerström G (1998) Parathyroid MEN1 gene mutations in relation to clinical characteristics of nonfamilial primary hyperparathyroidism. *J Clin Endocrinol Metab* **83**: 2960–2963

Chandrasekharappa SC, Guru SC, Manickam P, Olufemi S-E, Collins FS, Emmert-Buck MR, Debelenko LV, Zhuang Z, Lubenksy IA, Liotta LA, Crabtree JS, Wang Y, Roe BA, Weisemann J, Boguski MS, Agarwal SK, Kester MB, Kim YS, Heppner C, Dong Q, Spiegel AM, Burns AL and Marx SJ (1997) Positional cloning of the gene for multiple endocrine neoplasia-type 1. *Science* **276**: 404–407

Debelenko LV, Zhuang Z, Emmert-Buck MR, Chandrasekharappa SC, Manickam P, Guru SC, Marx SJ, Skarulis MC, Spiegel AM, Collins FS, Jensen RT, Liotta LA and Lubensky IA (1997a) Allelic deletions on chromosome 11q13 in multiple endocrine neoplasia type 1-associated and sporadic gastrinomas and pancreatic endocrine tumours. *Cancer Res* **57**: 2238–2243

Debelenko LV, Emmert-Buck MR, Zhuang Z, Epshteyn E, Moskaluk CA, Jensen RT, Liotta LA and Lubensky IA (1997b) The multiple endocrine neoplasia type 1 gene locus is involved in the pathogenesis of type 2 gastric carcinoids. *Gastroenterology* **113**: 773–781

Debelenko LV, Brambilla E, Agarwal SK, Swalwell JI, Kester MB, Lubensky IA, Zhuang Z, Guru SC, Manickam P, Olufemi S-E, Chandrasekharappa SC, Crabtree JS, Kim YS, Heppner C, Burns AL, Spiegel AM, Marx SJ, Liotta LA, Collins FS, Travis WD and Emmert-Buck MR (1997c) Identification of MEN1 gene mutations in sporadic carcinoid tumours of the lung. *Hum Mol Genet* **6**: 2285–2290

Debelenko LV, Emmert-Buck MR, Manickam P, Kester MB, Guru SC, DiFranco EM, Olufemi S-E, Agarwal SK, Lubensky IA, Zhuang Z, Burns AL, Spiegel AM, Liotta LA, Collins FS, Marx SJ and Chandrasekharappa SC (1997d) Haplotype analysis defines a minimal interval for the multiple endocrine neoplasia type 1 (MEN1) gene. *Cancer Res* **57**: 1039–1042

Dong Q, Debelenko LV, Chandrasekharappa SC, Emmert-Buck MR, Zhuang Z, Guru SC, Manickam P, Skarulis M, Lubensky IA, Liotta LA, Collins FS, Marx SJ and Spiegel AM (1997) Loss of heterozygosity at 11q13: analysis of piuitary tumors, lung carcinoids, lipomas, and other uncommon tumors with familial multiple endocrine neoplasia type 1. *J Clin Endocrinol Metab* **82**: 1416–1420

Farnebo F, Teh BT, Dotzenrath C, Wassif WS, Svensson A, White I, Betz R, Goretzki P, Sandelin K, Farnebo L-O and Larsson C (1997a) Differential loss of heterozygosity in familial, sporadic and uremic hyperparathryoidism. *Hum Genet* **99**: 342–349

Farnebo F, Farnebo L-O, Nordenström J and Larsson C (1997b) Allelic loss on chromosome 11 is uncommon in parathyroid glands of patients with hypercalcaemic secondary hyperparathyroidism. *Eur J Surg* **163**: 331–337

Farnebo F, Teh BT, Kytölä S, Svensson A, Phelan C, Sandelin K, Thompson NW, Höög A, Weber G, Farnebo L-O and Larsson C (1998) Alterations of the MEN1 gene in sporadic parathyroid tumors. *J Clin Endocrinol Metab* **83**: 2627–2630

Heppner C, Kester MB, Agarwal SK, Debelenko LV, Emmert-Buck MR, Guru SC, Manickam P, Olufemi S-E, Skarulis MC, Doppman JL, Alexander RH, Kim YS, Saggar SK, Lubensky IA, Zhuang Z, Liotta LA, Chandrasekharappa SC, Collins FS, Spiegel AM, Burns AL and Marx SJ (1997) Somatic mutation of the MEN1 gene in parathyroid tumours. *Nature Genet* **16**: 375–378

Iwasaki H, Stewart PW, Dilley WG, Holt MS, Steinbrueck TD, Wells SA and Donis-Keller H (1992) A minisatellite and a microsatellite polymorphism within 1.5 kb at the human muscle glycogen phosphorylase (PYGM) locus can be amplified by PCR and have combined informativeness of PIC 0.95. *Genomics* **13**: 7–15

Jakobovitz O, Nass D, DeMarco L, Barbosa AJA, Brok Simoni F, Rechavi G and Friedman E (1996) Carcinoid tumors frequently display genetic abnormalities involving chromosome 11. *J Clin Endocrinol Metab* **81**: 3164–3167

Landis CA, Masters SB, Spada A, Pace AM, Bourne HR and Vallar L (1989) GTPase inhibiting mutations activate the alpha chain of $G_s$ and stimulate adenyl cyclase in human pituitary tumours. *Nature* **340**: 692–696

Larsson C, Skogseid B, Öberg K, Nakamura Y and Nordenskjöld M (1988) Multiple endocrine neoplasia type 1 gene maps to chromosome 11 and is lost in insulinoma. *Nature* **332**: 85–87

Lemmens I, Van de Ven WJM, Kas K, Zhang CX, Giraud S, Wautot V, Buisson N, De Witte K, Salandre J, Lenoir G, Pugeat M, Calender A, Parente F, Quincey D, Gaudray P, De Wit MJ, Lips CJM, Höppener JWM, Khodaei S, Grant AL, Weber G, Kytölä S, Teh BT, Farnebo F, Phelan C, Hayward N, Larsson C, Pannett AAJ, Forbes SA, Bassett JHD and Thakker RV (1997a) Identification of the multiple endocrine neoplasia type 1 (MEN 1) gene. *Hum Mol Genet* **6**: 1177–1183

Manickam P, Guru SC, Debelenko LV, Agarwal SK, Olufemi S-E, Weisemann JM, Boguski MS, Crabtree JS, Wang Y, Roe BA, Lubensky IA, Zhuang Z, Kester MB, Burns AL, Spiegel AM, Marx SJ, Liotta LA, Emmert-Buck MR, Collins FS and Chandrasekharappa SC (1997) Eighteen new polymorphic markers in the multiple endocrine neoplasia type 1 (MEN1) region. *Hum Genet* **101**: 102–108

Miller SA, Dykes DD and Polesky HF (1988) A simple salting out procedure for extracting DNA from human nucleated cells. *Nucl Acids Res* **16**: 1215

Orita M, Suzuki Y, Sekiya Y and Hayashi K (1989) Rapid and sensitive detection of point mutations and DNA polymorphisms using the polymerase chain reaction. *Genomics* **5**: 874–879

© 2000 Cancer Research Campaign

Pack S, Turner ML, Zhuang Z, Vortmeyer AO, Böni R, Skarulis M, Marx SJ and Darling TN (1998) Cutaneous tumors in patients with multiple endocrine neoplasia type 1 show allelic deletion of the MEN1 gene. *J Invest Dermatol* **110**: 438–440

Prezant TR, Levine J and Melmed S (1998) Molecular characterization of the MEN1 tumor suppressor gene in sporadic pituitary tumors. *J Clin Endocrinol Metab* **83**: 1388–1391

Rosenberg CL, Kim HG, Shows TB, Kronenberg HM and Arnold A (1991) Rearrangement and overexpression of D11S287E, a candidate oncogene on chromosome 11q13 in benign parathyroid adenomas. *Oncogene* **6**: 449–453

Shan L, Nakamura Y, Nakamura M, Yokoi T, Tsujimoto M, Arima R, Kameya T and Kakudo K (1998) Somatic mutations of multiple endocrine neoplasia type 1 gene in the sporadic endocrine tumors. *Lab Invest* **78**: 471–475

Tahara H, Smith AP, Gaz RD, Cryns VL and Arnold A (1996) Genomic localisation of novel candidate tumor suppressor gene loci in human parathyroid adenomas. *Cancer Res* **56**: 599–605

Tanaka C, Yoshimoto K, Yamada S, Nishioka H, II S, Moritani M, Yamaoka T and Itakura M (1998) Absence of germline mutations of the multiple endocrine neoplasia type 1 (MEN1) gene in familial pituitary adenoma in contrast to MEN1 in Japanese. *J Clin Endocrinol Metab* **83**: 960–965

Teh BT, Hayward NK, Wilkinson S, Woods GM, Cameron D and Shepherd JJ (1990) Clonal loss of INT-2 alleles in sporadic and familial pancreatic endocrine tumours. *Br J Cancer* **62**: 253–254

Vortmeyer AO, Böni R, Pak E, Pack S and Zhuang Z (1998) Multiple endocrine neoplasia 1 gene alterations in MEN1-associated and sporadic lipomas. *J Natl Cancer Inst* **90**: 398–399

Woloschak M, Yu A, Xiao J and Post KD (1996) Frequent loss of the p16INK4a gene product in human pituitary tumours. *Cancer Res* **56**: 2493–2496

Zhuang Z, Vortmeyer AO, Pack S, Huang S, Pham TA, Wang C, Park W-S, Agarwal SK, Debelenko LV, Kester MB, Guru SC, Manickam P, Olufemi S-E, Yu F, Heppner C, Crabtree JS, Skarulis MC, Venzon DJ, Emmert-Buck MR, Spiegel AM, Chandrasekharappa SC, Collins FS, Burns AL, Marx SJ, Jensen RT, Liotta LA and Lubensky IA (1997a) Somatic mutations of the MEN1 tumor suppressor gene in sporadic gastrinomas and insulinomas. *Cancer Res* **57**: 4682–4686

Zhuang Z, Ezzat SZ, Vortmeyer AO, Weil R, Oldfield EH, Park W-S, Pack S, Huang S, Agarwal SK, Guru SC, Manickam P, Debelenko LV, Kester MB, Olufemi S-E, Heppner C, Crabtree JS, Burns AL, Spiegel AM, Marx SJ, Chandrasekharappa SC, Collins FS, Emmert-Buck MR, Liotta LA, Asa SL and Lubensky IA (1997b) Mutations of the MEN1 tumor suppressor gene in pituitary tumors. *Cancer Res* **57**: 5446–5451

 *© 2000 Cancer Research Campaign*
